# Recurrent laryngeal nerve palsy due to impacted dental plate in the thoracic oesophagus: case report

**DOI:** 10.1186/1749-7922-2-30

**Published:** 2007-11-12

**Authors:** Robert P Sutcliffe, Ashish Rohatgi, Matthew J Forshaw, Robert C Mason

**Affiliations:** 1 Department of General Surgery, Guy's and St Thomas' NHS Trust, St Thomas' Hospital, Lambeth Palace Road, London SE1 7EH, UK

## Abstract

**Background:**

Retained oesophageal foreign bodies must be urgently removed to prevent potentially serious complications. Recurrent laryngeal nerve palsy is rare and has not been reported in association with a foreign body in the thoracic oesophagus.

**Case presentation:**

We present a case of a dental plate in the thoracic oesophagus that caused high dysphagia. Delayed diagnosis led to a recurrent laryngeal nerve palsy, which persisted despite successful surgical removal of the foreign body.

**Conclusion:**

Oesophagoscopy is essential to fully assess patients with persistent symptoms after foreign body ingestion, irrespective of the level of dysphagia. Recurrent laryngeal nerve palsy may indicate impending perforation and should prompt urgent evaluation and treatment.

## Background

Retained oesophageal foreign bodies may cause potentially fatal complications such as perforation or fistula, and must be removed urgently [1-4]. The majority of cases are successfully removed by either rigid or flexible oesophagoscopy, whilst surgery is rarely indicated [5]. We report a case of an impacted dental plate in the thoracic oesophagus presenting with recurrent laryngeal nerve palsy that was safely removed by thoracotomy one year after ingestion.

## Case presentation

**Figure 1:**
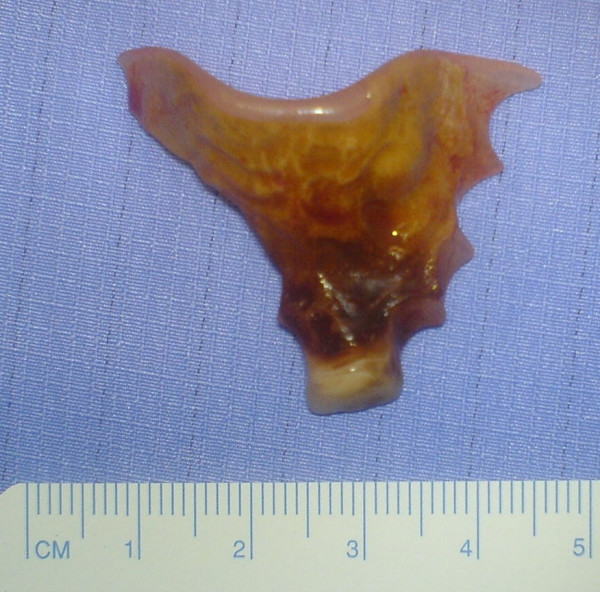
Dental plate removed from oesophagus via transthoracic oesophagotomy.

**Figure 2:**
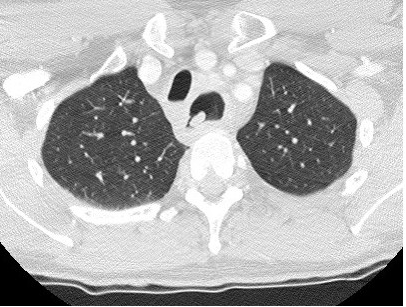
Oesophageal thickening and intraluminal mass seen on computed tomograghy (lung windows).

**Figure 3:**
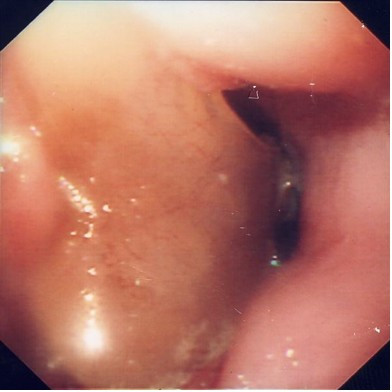
Dental plate impacted in oesophagus at endoscopy.

**Figure 4:**
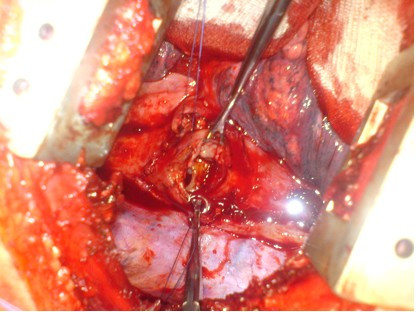
Transthoracic view of dental plate impacted in oesophagus.

A 42-year-old man presented with a 4-month history of hoarse voice and a 12-month history of intermittent high dysphagia and odynophagia. His symptoms started immediately after he accidentally swallowed an acrylic dental plate (Figure 1) whilst eating. He presented to the emergency department of his local hospital: plain x-rays were performed and reported as normal. He was reassured and discharged. Over the next 6 months, his symptoms of high dysphagia and odynophagia persisted. His general practitioner referred him to the local ENT department. Flexible laryngoscopy was normal on two occasions. A barium swallow was also reported as normal. Two months later, after swallowing a piece of meat, he had an episode of complete dysphagia followed by severe odynophagia and a hoarse voice. Repeat flexible laryngoscopy demonstrated left recurrent laryngeal nerve palsy and he was referred for urgent computed tomography of the neck and chest. This showed thickening in the mid-oesophagus and a possible intraluminal foreign body (Figure 2). At oesophagoscopy, the dental plate was found to be impacted in the oesophagus at the level of the aortic arch (25 cm from the incisor teeth; Figure 3) and could not be safely removed. He was referred to a specialist oesophagogastric unit for surgical intervention. Due to its location in the thoracic oesophagus, it was elected to approach the foreign body via a right posterolateral thoracotomy through the fourth intercostal space. At operation, the mid-oesophagus appeared thickened, and there was marked peri-oesophageal inflammation and fibrosis. There was no evidence of perforation or fistula within the right pleural cavity. After dividing the azygos vein, the dental plate was removed via a longitudinal oesophagotomy (Figure 4), which was closed in two layers with an absorbable 2/0 suture. He made an uneventful postoperative recovery. A postoperative water-soluble contrast study was normal, and he was discharged on day 8. At follow up, his swallowing had returned to normal, but he had a persistent hoarse voice due to recurrent laryngeal nerve palsy, for which he has been referred for injection laryngoplasty.

## Discussion

Prompt diagnosis and retrieval of impacted foreign bodies in the oesophagus is essential to avoid potentially fatal complications such as perforation and fistulation into surrounding structures such as the tracheobronchial tree or aorta [1-4]. This case illustrates the difficulties of confirming the presence of a retained oesophageal foreign body despite a strong clinical suspicion and the increased symptomatic and surgical morbidity that resulted from such a delayed diagnosis.

A number of investigations can help in diagnosing a retained oesophageal foreign body. Plain radiography can diagnose impacted radio-opaque objects, but is of limited value in detecting radiolucent objects [6]. Contrast studies may also be normal, as in this patient, but are possibly contraindicated due to risk of aspiration [7]. Flexible laryngoscopy allows diagnosis and removal of foreign bodies in the hypopharynx and proximal oesophagus. However, negative laryngoscopy should prompt further assessment by flexible oesophagoscopy to inspect the entire oesophagus [8]. Oesophagoscopy is the investigation of choice for evaluating persistent oesophageal symptoms in any patient with a history of foreign body ingestion. In this case, failure to perform oesophagoscopy resulted in delayed diagnosis and led to the development of a recurrent laryngeal nerve palsy.

The differential diagnosis of recurrent laryngeal nerve palsy includes malignant, traumatic, inflammatory and neurological processes [9]. Recurrent laryngeal nerve palsy has been reported rarely in relation to impacted foreign bodies in the hypopharynx or cervical oesophagus [10-13], and has not been reported previously in relation to a foreign body in the thoracic oesophagus. In this patient, peri-oesophageal inflammation due to the dental plate caused left recurrent laryngeal nerve compression in the region of the aortic arch, and probably reflected imminent perforation or fistulation.

Oesophageal foreign bodies usually impact at the level of cricopharyngeus or at the aortic arch, and should be removed urgently to prevent complications [7]. Most foreign bodies can be safely removed by flexible oesophagoscopy with an overtube using various devices (for example, forceps, snares, dormia basket, Roth net^®^) [14]. If flexible oesophagoscopy is unsuccessful, rigid oesophagoscopy may be performed under general anaesthesia. Balloon extraction of foreign bodies under fluoroscopic guidance has also been reported, but this technique is not widely practised [14].

If endoscopy is unsuccessful, surgical removal is indicated and should be performed by an experienced surgeon in a specialist centre. The first recorded case of surgical removal of an oesophageal foreign body was by Grey Turner in 1947 [15]. A cervical or transthoracic oesophagotomy is usually sufficient although the exact surgical approach will depend upon the level of obstruction. Rarely, oesophageal diversion and/or oesophagectomy may be necessary in the presence of significant oesophageal injury, fistula or contamination [1].

## Conclusion

This case illustrates the importance of flexible oesophagoscopy in assessing patients with persistent oesophageal symptoms after foreign body ingestion, irrespective of the level of dysphagia. Recurrent laryngeal nerve palsy is rare in these patients, but its presence should prompt urgent evaluation and treatment to avoid potentially serious complications.

## Competing interests

The author(s) declare that they have no competing interests.

## Authors' contributions

RS and MF collected the data and drafted the manuscript. AR performed a literature review and helped draft the manuscript. RM conceived the study. All authors have read and approved the final manuscript.
